# Disease surveillance, capacity building and implementation of the International Health Regulations (IHR[2005])

**DOI:** 10.1186/1471-2458-10-S1-S1

**Published:** 2010-12-03

**Authors:** Rebecca L Katz, Jose A Fernandez, Scott JN McNabb

**Affiliations:** 1 U.S. Department of State, Washington, DC, USA; 2 George Washington University, School of Public Health and Health Services, Washington, DC, USA; 3 U.S. Department of Health and Human Services, Washington, DC, USA; 4 Emory University, Atlanta, GA, USA

## Introduction

At the December 2009 Meeting of States Parties of the Biological Weapons Convention (BWC), U.S. Under Secretary of State Ellen Tauscher committed the U.S. Government (USG) to engaging the global community to achieving and sustaining the capabilities to combat infectious diseases and protect against biological threats. Specifically, she committed the USG to a series of actions, including international meetings on global disease surveillance and implementation of the International Health Regulations (IHR[2005]), designed to enhance global cooperation and provide momentum for sustained progress in this critical area. This journal supplement includes articles that capture key presentations from two meetings: the June 2010 workshop on Comprehensive Global Disease Surveillance held in Washington, D.C. and the August 2010 workshop on Implementation of the IHR(2005) held in Geneva, Switzerland. The supplement also highlights efforts underway to enhance disease surveillance and IHR(2005) implementation by global partners and frames the current USG efforts to enhance global cooperation in disease surveillance, capacity building, biothreat reduction, and IHR(2005) implementation.

The IHR(2005) provides a framework to promote global health security in the broadest sense. Public health emergencies of international concern (PHEICs), by definition, do not respect international boundaries, and the IHR(2005) articulates a vision of solidarity that a common vulnerability to microbial and other threats should elicit. A common interest exists for all countries to possess the capacities and capabilities identified in the IHR(2005) to detect, assess, report, and respond to public health threats, whether they are naturally occurring, accidental, or deliberate in origin. This interest is neither solely a public health interest, nor a security interest, but a human interest. Accordingly, the public health and security communities have found it increasingly beneficial to work together to advance their shared objectives in this particular area. While these two communities operate in distinct spheres, there is an area where the public health and security spheres overlap. These workshops brought the two communities together to clarify the connections between these spheres and to promote and enhance cooperative efforts between them to advance IHR(2005) implementation internationally in an effective, meaningful, and sustainable manner.

## Working towards comprehensive global disease surveillance

On June 16^th^ and 17^th^, 2010 more than 140 health and security experts from 30 countries gathered in Washington, D.C. to discuss the fundamental components of comprehensive disease surveillance, impediments to implementing efficient and effective systems, and lessons and recommendations under the IHR(2005) that help build core disease surveillance capacity. The meeting identified policy imperatives necessary to achieve functional, comprehensive systems, particularly in low-resource settings and provided a venue for funders and aid recipients to discuss the core capacities for surveillance, as outlined in Annex 1 of the IHR(2005).

**Figure 1:**
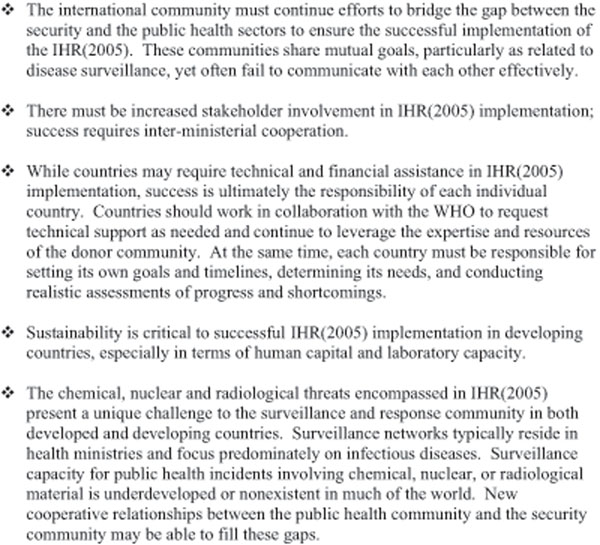
Key themes from breakout sessions at the Workshop on Moving Towards Comprehensive Global Disease Surveillance.

The June workshop included presentations from U.S. senior officials from Department of State (DoS), Department of Health and Human Services (DHHS), and the Department of Defense (DoD). The U.S. National Security Staff highlighted the political-level commitment for increased coordination between the health and security communities. Representatives from across the USG described their agencies’ efforts to build global disease surveillance capacity, and global experts gave overviews of essential components of effective surveillance; including human workforce development, commu-nications, epidemiologic capacity, and the human/animal interface. The remainder of the workshop was spent in break-out sessions, enabling participants to share national viewpoints, experiences, and suggestions for cooperative efforts (see Figure 1).

In this journal supplement, we include six articles drawn directly from this workshop. Drs. McNabb and Chungong provide an overview of global surveillance elements, the important scientific, political, and technologic drivers of public health surveillance, and the surveillance core capacities required for compliance with the IHR(2005). Drs. Kant and Krishnan describe how information and communication technology is being used for disease surveillance in India. Mr. Johns and Dr. Blazes discuss how the Department of Defense is helping nations building core capacities for IHR(2005). Dr. Nsubuga from the Centers for Disease Control and Prevention (CDC), along with colleagues from the U.S. Agency for International Development (USAID), the Africa Field Epidemiology Network (AFENET) and CDC present mechanisms for strengthening surveillance and response capacity using the health systems strengthening agenda for developing countries. Dr. Andrus and colleagues from the Pan American Health Organization (PAHO) write about global health security in the context of the IHR(2005), with specific examples of how IHR(2005) guided the response to yellow fever in Paraguay and the H1N1 pandemic. Also in this supplement is the overview of the USG agencies and offices engaged in building global capacity for disease surveillance, as representatives presented it at this meeting.

## Implementation of the IHR(2005)

On August 20^th^, 2010 a follow-on workshop was held at the Palais des Nations in Geneva, Switzerland co-hosted by the BWC Implementation Support Unit. This workshop again brought more than 100 experts from around the world together for detailed discussion of lessons learned from national experiences implementing the IHR(2005) and regional efforts to support capacity building. The aim of this workshop was to share insights into the practical implementation of the IHR(2005), to identify and address obstacles, and to facilitate sustainable, long-term collaborations. Speakers representing four WHO regions delivered national presentations, including Uganda, represented in the article by Wamala, *et al*. WHO representatives spoke about international collaboration efforts necessary for IHR(2005) implementation and representatives from the AFENET and the American Society for Microbiology (ASM) spoke about capacity building efforts. These presentations are represented by articles by Dr. Specter and colleagues from ASM, and by Dr. Musenero and colleagues from AFENET.

**Figure 2:**
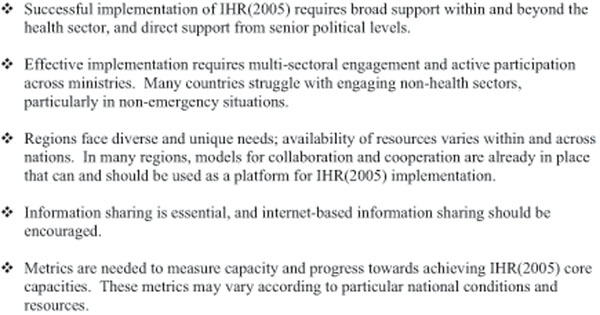
Key themes, concerns and suggestions from participants at the Meeting on Implementation of the IHR(2005).

Several major themes emerged from the meeting (see Figure 2), as well as specific challenges identified by participants. Some of the specific challenges to successful IHR(2005) implementation include:

• Some countries struggle with gaps in resources, particularly human resources. Participants emphasized the importance of regional training centers to address workforce shortages and training gaps.

• Meeting IHR(2005) obligations at Points of Entry is a universal challenge, involving human resources and multi-sectoral engagement and communication.

• The safe and effective transportation of specimens and samples remains difficult in many parts of the world.

• There is a need for better laboratory infrastructure. Specifically, labs need broad spectrum diagnostics for rare diseases and common reagents.

• Some countries have had success in developing core capacities at the national level, but found it challenging to make substantial progress in developing capacity at the local level.

• Some countries are focused on building basic public health infrastructure to address endemic health needs, and must prioritize developing this basic infrastructure before focusing specifically on IHR(2005) compliance.

Workshop participants discussed a set of eight draft principles for capacity building and global cooperation for implementing IHR(2005). They include:

1. In today’s interdependent and interconnected world, health security requires coordinated action and cooperation among members of the international community.

2. No single institution or country has all the capacities needed to effectively respond to international public health emergencies. An effective response to these events requires cooperation among multiple sectors and multiple partner countries, as well as the WHO.

3. The IHR(2005) provide a critical and universal framework for promoting global health security.

4. Early detection, rapid reporting, and effective response are critical to prevent or halt the international spread of disease.

5. Rapid and timely communications between countries and with the WHO is critical for the response to international public health emergencies.

6. Strong health systems are critical to each country’s ability to prepare for and respond to both routine public health events and public health emergencies with international impact.

7. Capacity-building must be practical, sustainable, collaborative, and based on the needs of each country. In this regard, these efforts must contribute to the strengthening of each country’s day-to-day capacities to detect and respond to public health events.

8. The development and maintenance of the IHR(2005) core capacities require a significant investment on the part of all countries. To maximize the effectiveness and efficiency of these capacity-building activities, it is important to take full advantage of opportunities for collaboration and coordination among partners.

## Additional policy issues

Several active participants in the summer meetings have articles in this supplement relevant to IHR(2005), disease surveillance and capacity building. Dr. Bakanidze from Georgia and her co-authors write about biosafety and biosecurity as pillars of international health security, and discuss how Georgia is building a biosafety regime using the international guidelines provided by IHR(2005), the BWC and United Nations Security Council Resolution 1540. Dr. Sobers and her colleagues from Barbados detail the island nation’s experience with H1N1 and the actions taken by the government to mitigate the consequences of the disease on their country. Finally, colleagues from the CDC the Defense Threat Reduction Agency (DTRA) collaborate on a paper that provides a systems approach to strengthening national surveillance and detection of events of public health importance.

## Conclusions

Representing the desire to foster global collaboration and find both a common political and technical vision for full implementation of the IHR(2005), the representatives at the June and August meetings, as well as a growing network of international partners are achieving important consensus, activities, and outputs. Countries recognize gaps in disease surveillance capacity and needs for intra-country and inter-sector collaboration. They also face challenges in specific technical areas and in building leadership, communication, and collaboration. The platform for discussion and planning provided in June and August generated enthusiasm and targeted areas for intervention. The contributors to this supplement are codifying the vision for global disease surveillance and IHR(2005) implementation, and collectively, planning the future.

## Abbreviations

AFENET: Africa Field Epidemiology Network; BWC: Biological Weapons Convention; CDC: Centers for Disease Control and Prevention; DHHS: Department of Health and Human Services; DoD: Department of Defense; DoS: Department of State; DTRA: Defense Threat Reduction Agency; IHR: International Health Regulations; PAHO: Pan American Health Organization; PHEIC: Public health emergency of international concern; USAID: United State Agency for International Development; USG: United States Government.

## Competing interests

No competing interests to declare.

## Authors’ contributions

All authors contributed equally to the text.

